# The Novel Non-coding Transcriptional Regulator Gm18840 Drives Cardiomyocyte Apoptosis in Myocardial Infarction Post Ischemia/Reperfusion

**DOI:** 10.3389/fcell.2021.615950

**Published:** 2021-07-12

**Authors:** Changjun Luo, Si Xiong, Yiteng Huang, Ming Deng, Jing Zhang, Jianlin Chen, Rongfeng Yang, Xiao Ke

**Affiliations:** ^1^Afficiated Liutie Central Hospital & Clinical Medical College of Guangxi Medical University, Guangxi, China; ^2^Department of Cardiology, Fuwai Hospital, Chinese Academy of Medical Sciences, Shenzhen, (Shenzhen Sun Yat-sen Cardiovascular Hospital), Shenzhen, China; ^3^Key Laboratory of Biomaterials of Guangdong Higher Education Institutes, Department of Biomedical Engineering, Jinan University, Guangzhou, China; ^4^Shenzhen University School of Medicine & Shenzhen University Health Science Center, Shenzhen, China

**Keywords:** myocardial infarction, transcriptional regulation, lncRNA, Gm18840, cardiomyocytes apoptosis

## Abstract

**Background:**

Ischemia/reperfusion-mediated myocardial infarction (MIRI) is a major pathological factor implicated in the progression of ischemic heart disease, but the key factors dysregulated during MIRI have not been fully elucidated, especially those essential non-coding factors required for cardiovascular development.

**Methods:**

A murine MIRI model and RNA sequencing (RNA-seq) were used to identify key lncRNAs after myocardial infarction. qRT-PCR was used to validate expression in cardiac muscle tissues and myocardial cells. The role of Gm18840 in HL-1 cell growth was determined by flow cytometry experiments *in vitro*. Full-length Gm18840 was identified by using a rapid amplification of cDNA ends (RACE) assay. The subcellular distribution of Gm18840 was examined by nuclear/cytoplasmic RNA fractionation and qRT-PCR. RNA pulldown and RNA immunoprecipitation (RIP)-qPCR assays were performed to identify Gm18840-interacting proteins. Chromatin isolation by RNA purification (ChIRP)-seq (chromatin isolation by RNA purification) was used to identify the genome-wide binding of Gm18840 to chromatin. The regulatory activity of Gm18840 in transcriptional regulation was examined by a luciferase reporter assay and qRT-PCR.

**Results:**

Gm18840 was upregulated after myocardial infarction in both *in vivo* and *in vitro* MIRI models. Gm18840 was 1,471 nt in length and localized in both the cytoplasm and the nucleus of HL-1 cells. Functional studies showed that the knockdown of Gm18840 promoted the apoptosis of HL-1 cells. Gm18840 directly interacts with histones, including H2B, highlighting a potential function in transcriptional regulation. Further ChIRP-seq and RNA-seq analyses showed that Gm18840 is directly bound to the *cis*-regulatory regions of genes involved in developmental processes, such as Junb, Rras2, and Bcl3.

**Conclusion:**

Gm18840, a novel transcriptional regulator, promoted the apoptosis of myocardial cells via direct transcriptional regulation of essential genes and might serve as a novel therapeutic target for MIRI.

## Introduction

Myocardial infarction (MI) is a major cause of morbidity and mortality (Hausenloy et al., 2013; Reed et al., 2017) and affects more than 50 million people worldwide (Lopez et al., 2006). MI usually results in cardiomyocytes suffering from hypoxia, which induces a severe inflammatory response, promotes myocyte apoptosis and cardiac fibrosis, and finally leads to worse cardiac function and even heart failure (Matyas et al., 2017; Zhang et al., 2017; Ong et al., 2018). Thus, identifying key dysregulated programs and factors during MI is of great importance for the development of potential novel therapeutic strategies.

Emerging evidence has demonstrated that long non-coding RNAs (lncRNAs) display diverse cellular functions and widely participate in both physiological and pathophysiological processes (Li and Sun, 2020; Sayad et al., 2020; Zhang et al., 2020). LncRNAs are a diverse class of RNA transcripts longer than 200 nucleotides that have limited protein coding capacity (Zhou et al., 2020). Most lncRNAs do not engage in translation but exert critical roles in multiple regulatory functions, including microRNA sponging, transcriptional regulation, translational regulation, and protein–protein interactions. Notably, the transcriptional regulatory roles of lncRNAs have emerged as major functions in many key developmental processes. These lncRNAs can function by directly binding to histone-associated complexes, transcription factors, coregulators, or RNA polymerase II within distinct *cis*-regulatory regions.

Recently, lines of evidence have indicated that lncRNAs also play essential roles in the occurrence and development of cardiovascular diseases (Viereck et al., 2016; Kenneweg et al., 2019; Lu et al., 2019; Yang et al., 2019). For example, lncRNA MIAT is involved in the regulation of MI through the regulation of the ERK/MAPK signaling pathway (Fan et al., 2019). Zhang D. et al. (2019) reported that lncRNA HOTAIR protects MI rats by sponging miR-519d-3p. Wang et al. (2015) found that lncRNA AFP regulates MI by targeting miR-188-3p. Moreover, lncRNAs SLC8A1-AS1 (Guo G. L. et al., 2019), ZFAS1 (Jiao et al., 2019), and GAS5 (Zhang J. C. et al., 2019) play regulatory roles in the occurrence and development of MI in different ways. However, lncRNAs acting as non-coding transcriptional regulators in MI are rarely reported.

In this study, we identified a new lncRNA, Gm18840, as a non-coding transcriptional regulator that was significantly upregulated in MI myocardial tissue compared with the normal myocardial tissue of mice. With a combination of RNA sequencing (RNA-seq), bioinformatic analysis, and several molecular and cellular biology experiments, we elucidated novel mechanisms of MI.

## Materials and Methods

### MI Mouse Model

Animal experiments were approved by the Animal Care and Use Committee of Affiliated Liutie Central Hospital & Clinical Medical College of Guangxi Medical University. C57BL/6 male mice (8 weeks old) were obtained from Shanghai SLAC Laboratory Animal Co., Ltd. (Shanghai, China), fed under specific pathogen-free conditions, and randomly treated with MI. To anesthetize the mice, 50 μg/g body weight ketamine and 60 μg/g body weight sodium pentobarbital were injected intraperitoneally into the mice (*n* = 7). Afterward, the mice were intubated and placed onto a rodent ventilator. A 5-mm incision was cut between the fourth and fifth intercostal spaces of the chest cavity. The left-anterior descending coronary artery (LAD) was observed by a microscope, followed by ligation using a 7-0 Prolene suture. Mice in the sham group were subjected to the same procedure without suture but with a moving suture behind the LAD artery. Finally, CO_2_ asphyxiation was used to anesthetize and euthanize the mice to collect the hearts.

### Cell Culture

The mouse cardiomyocyte cell line HL-1 was purchased from the American Type Culture Collection (ATCC) and cultured in a Claycomb medium (51800C, Signal-Aldrich) with 10% FBS, 0.1 mM norepinephrine, 2 mM L-glutamine, and antibiotics (100 U/ml penicillin, 100 μg/ml streptomycin). Cells were maintained in a humidified incubator (Thermo Fisher Scientific) at 37°C with 5% CO_2_.

### Establishment of an Ischemia/Reperfusion Cell Model

HL-1 cells were grown to 80% confluence and maintained in a quiescent state for 12 h. To generate hypoxic conditions, cells were transferred to an incubation chamber (Billups-Rothenberg MIC-101) and flushed with a hypoxic gas mixture (95% N2, 5% CO_2_) for 24 h. Subsequently, the cells were subjected to reoxygenation treatment at 37°C for 2 h to induce the establishment of an ischemia/reperfusion (I/R) model.

### RNA Isolation and Quantitative Reverse Transcription PCR (qRT-PCR)

Total RNA was extracted with a TRIzol reagent (Life Technologies) according to the instructions of the manufacturer. qRT-PCR was performed using an AceQ Universal SYBR qPCR Master Mix (Vazyme) according to the protocols of the manufacturer. The relative expression of genes was normalized to GAPDH or U6 and expressed as fold change compared with the internal standard via the 2^–ΔΔCT^ method in this study. The primers are shown in Table 1.

**TABLE 1:** Specific primer sequence of genes.

**Gene symbol**	**Sequences (5′–3′)**
Gm18840	F: CTGGAAGCTGCACTACACCA
	R:TGAAGTAATCCCGGAACTGG
Dnm3os	F: GTGTTGGACAGAAGCACACG
	R:TGTGCAGTGCCTAGAGATGG
Meg3	F:TCCTCACCTCCAATTTCCCCT
	R:GAGCGAGAGCCGTTCGATG
Pvt1	F:TCGTGATGGGTTCCGTAGAGG
	R:GCTGGGATGCACATTTCTCTG
GAPDH	F:AGGTCGGTGTGAACGGATTTG
	R:TGTAGACCATGTAGTTGAGGTCA
U6	F:ACCCTGAGAAATACCCTCACAT
	R:GACGACTGAGCCCCTGATG

### Apoptosis Analysis

To determine myocardium apoptosis levels, cultured HL-1 cells were collected after transfection and rinsed in chilled PBS, followed by double staining with an Annexin V fluorescein isothiocyanate (FITC)/propidium iodide (PI) detection kit (Invitrogen) in the dark for 15 min. Then, the rates of apoptotic cells were detected by a flow cytometer (Beckman Coulter, Brea, CA, United States).

### Rapid Amplification of cDNA Ends

The 5′- and 3′-rapid amplification of cDNA ends (RACE) experiments were performed using the SMARTer RACE Kit (Clontech) according to the instructions of the manufacturer. The RACE PCR products were separated on an agarose gel. PCR bands were cloned into the pGM-T vector (TIANGEN Biotech, China) and sequenced. The RACE primers targeting Gm18840 used for the nested PCR are listed below: 5′-RACE outer primer, 5′-CCATGTGCCATCATGCTGGCAAA-3′; 5′-RACE inner primer-1, 5′-CTGGTAGCCTTGCTTGCAGGTGACA-3′; 3′-RACE outer primer, 5′-CCTGCAAGCAAGGCTACCAGCTCAT-3′; and 3′-RACE inner primer, 5′-ATGCCCAGGTGCAAGAT CAAGAACTGTG-3′.

### RNA Interference

All small interfering RNAs (siRNAs) against Gm18840 were purchased from Invitrogen (United States). The target sequences were as follows: sense 5′-GUUCCGGGAUU ACUUCAUUGUTT-3′ and antisense 5′-ACAAUGAAGUAA UCCCGGAACTT-3′. HL-1 cells were transiently transfected with siRNAs using Lipofectamine 2000 (Invitrogen) following the instructions of the manufacturer.

### RNA Sequencing

RNA was extracted using a TRIzol reagent (Life Technologies) according to the instructions of the manufacturer. The RNA-seq library was constructed using the VAHTS mRNA-seq V2 Library Prep Kit for Illumina^®^ (NR601, Vazyme) according to the instructions of the manufacturer. Sequencing was performed using Illumina’s NovaSeq platform according to the instructions of the manufacturer. We have uploaded the RNA-seq data to a public dataset, SAR (PRJNA669226).

### RNA Pulldown Assay

RNA pulldown assays were performed using the Pierce^TM^ Magnetic RNA-Protein Pull-Down Kit (#20164, Thermo Fisher Scientific, United States) according to the instructions of the manufacturer. Full-length Gm18840 RNA was obtained using *in vitro* transcription with the T7 High Yield RNA Transcription Kit (TR101, Vazyme), after which the RNA was biotinylated using the Pierce^TM^ RNA 3′ End Desthiobiotinylation Kit (# 20163, Thermo Fisher Scientific, United States). Cell extracts were incubated with RNA for 20 min, followed by the addition of nucleic acid-compatible streptavidin magnetic beads (Thermo Fisher Scientific) for further incubation. After washing five times, Gm18840-associated proteins, which were retrieved from beads, were subjected to SDS-PAGE and silver staining. Subsequently, the protein bands were excised and identified by liquid chromatography–mass spectrometry (LC-MS/MS).

### RNA Immunoprecipitation Assay

The RNA Immunoprecipitation (RIP) assay was conducted with the Magna RIP^TM^ RNA-Binding Protein Immunoprecipitation Kit (Millipore, United States) according to the instructions of the manufacturer. Briefly, cells were lysed in a lysis buffer containing a protease inhibitor cocktail and an RNase inhibitor. Then, cell extracts were incubated with magnetic beads conjugated with control IgG or anti-Tim50 antibody (Proteintech Group, China). Immunoprecipitated RNAs were purified and quantified by qRT-PCR.

### Luciferase Reporter Analysis

The sequences of Gm18840 and Junb, Rras2, and Bcl3 3′-UTRs were amplified using qRT-PCR and inserted into the pGL3 luciferase vector (Promega). HL-1 cells were transfected with a firefly luciferase vector (100 ng) and a Renilla luciferase expression vector (10 ng). Cells were transiently cotransfected with luciferase-reporter plasmids (Promega) with Lipofectamine 2,000 according to the instructions of the manufacturer. After cotransfection with the indicated plasmids for 48 h, the Dual-Luciferase Reporter Assay System (Promega) was used. Renilla luciferase (pRL-TK) acted as an internal control to normalize transfection efficiencies.

### Chromatin Isolation by RNA Purification

Chromatin isolation by RNA purification (ChIRP) was performed as in a previous study (Chu et al., 2012). Gm18840-transduced HL-1 cells were fixed with 1% formaldehyde for 10 min and quenched in 125 mmol/L glycine for 5 min. The cells were collected and washed twice with PBS and then resuspended in a sonication buffer (20 mmol/L Tris–HCl (pH 8), 2 mmol/L EDTA, 1% Triton X-100, 150 mmol/L NaCl, 1% SDS). The samples were sonicated using the Bioruptor^®^ PicoSonication System (Diagenode, Belgium) for 10 cycles (30 s on/30 s off). After centrifugation for 10 min at 15,000 *g* at 4°C, the supernatants were collected and incubated with biotinylated antisense DNA against Gm18840 at 4°C overnight. The probes used in the ChIRP assay are listed as follows: Gm18840-1: 5′-TTGCTGCTGGTGTCAGGTCT-3′; GM18840-2: 5′-TGCACGATGCCATGTGCCAT-3′; GM18840-3: 5′-TTCAGGGCTGCCCAAAGTTC-3′; Gm18840-4: 5′-TA CCTCACAGGGACTCGCTG-3′. The antisense probes for each DNA sequence were used as negative controls. Streptavidin magnetic beads were washed and added to the reaction mixture for 4 h at 4°C. The beads were washed five times with a wash buffer (20 mmol/L Tris–HCl (pH 8), 2 mmol/L EDTA, 1% Triton X-100, 300 mmol/L NaCl, 0.2% SDS). The ChIRPed samples were eluted using a biotin elution buffer and decrosslinked with Proteinase K at 65°C overnight. ChIRPed DNA was purified using the QIAquick PCR Purification Kit (Qiagen).

### Bioinformatic Analysis

RNA sequencing libraries were constructed according to the instructions of the manufacturer for the VAHTS Total RNA-seq (H/M/R) Library Prep Kit for Illumina (Vazyme, China). The libraries were sequenced on the Illumina Nova-seq 6000 platform. The raw sequenced reads were mapped to the mouse genome (mm10) using the STAR software (Dobin et al., 2013). Genes were annotated using the Ensemble database. The expression of the genes was quantified using HTseq (Anders et al., 2015), and the differentially expressed genes (DEGs) were identified using DEseq2 (Love et al., 2014). Genes with FDR < 0.01 and | Log_2_ (fold change)| > 1 were considered DEGs. Gene ontology (GO) analysis was performed using the DAVID database (Huang da et al., 2009). For the ChIRP-seq analysis, raw reads were mapped to the mouse genome (mm10) using the bowtie2 software (Langmead and Salzberg, 2012). Peaks were called with the MACS suite (Zhang et al., 2008). Motif enrichment analysis was performed using the MEME suite (Bailey et al., 2015).

### Statistical Analysis

Statistical analyses were performed using SPSS v.23.0 or Prism GraphPad 8.0. For most of the experiments, independent sample *t*-tests were used to calculate the p values. The experiments, with representative data shown in the figures, were repeated three times independently with similar results obtained. Statistical details and methods used are indicated in the figure legends, text, or methods.

## Results

### Identification of lncRNAs That Are Differentially Expressed Upon Myocardial Ischemia-Reperfusion Injury

**FIGURE 1:**
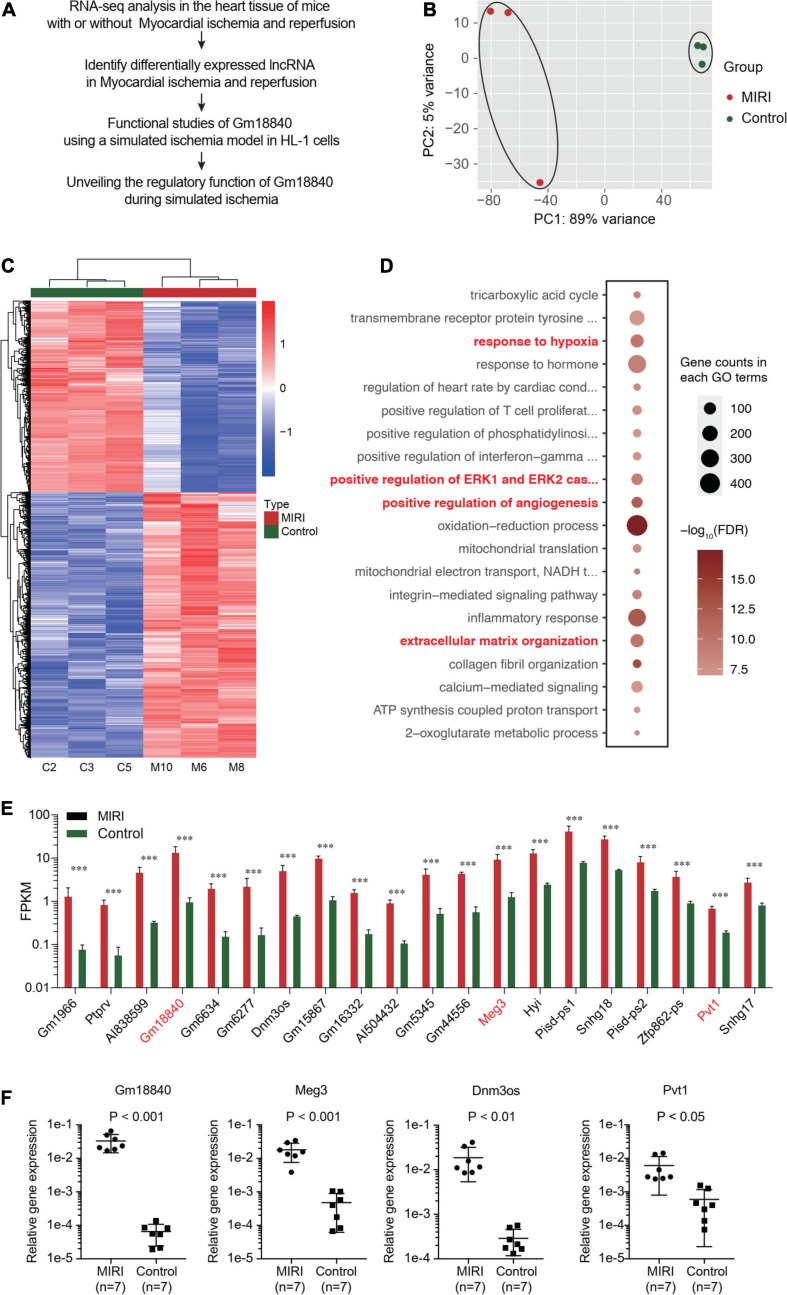
Identify lncRNAs that are differentially expressed upon myocardial ischemia–reperfusion injury. **(A)** The workflow of experimental design. Overall view of myocardial ischemia–reperfusion injury-related lncRNA identification and functional and mechanistic analysis of Gm18840 in myocardial ischemia–reperfusion injury. **(B)** Distinct gene expression patterns in cardiac tissues with or without IRI treatment. PCA plot showing the clustering of the RNA expression profile of cardiac tissue in mice with or without IRI. The red dots represent the IRI samples, and the blue dots represent the normal samples. **(C)** Heatmap showing differentially expressed genes in cardiac tissues with or without IRI. Detailed differentially expressed genes are provided in Supplementary Tables 1, 2,. **(D)** Gene ontology analysis of differentially expressed genes in cardiac tissues with or without IRI treatment. **(E)** Illustration of differentially expressed lncRNAs in cardiac tissues upon IRI treatment. **(F)** Validation of the differentially expressed lncRNAs by qRT-PCR in cardiac tissues with or without IRI treatment. ***p < 0.001.

To identify key protein coding and non-coding genes related to myocardial ischemia–reperfusion injury, we performed transcriptomic analysis in myocardial tissues of mice with or without ischemia/reperfusion injury (IRI). As shown in Figure 1A, the cardiac tissues were dissected 7 days post-IRI treatment, and RNA-seq analysis was performed in the heart tissue of mice with or without myocardial ischemia–reperfusion injury (MIRI). The general quality of the RNA-seq experiments is shown in Supplementary Figure 1. A total of 1,936 upregulated protein-coding genes, 2,731 downregulated protein-coding genes, 356 upregulated non-coding genes, and 303 downregulated non-coding genes were identified (FDR < 0.01 and | Log_2_(Fold change)| > 1) (Supplementary Tables 1, 2). Differentially expressed protein-coding genes and lncRNAs were identified. A principal component analysis (PCA) plot showed the clustering of the RNA expression profiles of cardiac tissues in mice with or without IRI (Figure 1B). In addition, differentially expressed genes in cardiac tissues with or without IRI are shown in the heatmap (Figure 1C). Figure 1E illustrates the differentially expressed lncRNAs in cardiac tissues upon IRI treatment. GO functional enrichment analysis of these differentially expressed genes in cardiac tissues with or without IRI treatment showed that genes related to development processes, oxidation reduction processes, and anatomical structure development were significantly enriched (Figure 1D and Supplementary Table 3), supporting the observed ischemia/reperfusion injury in heart tissues. Four lncRNAs, Gm18840, Meg3, Dnm3os, and Pvt1, were shown to be the most differentially expressed in cardiac tissues with or without IRI. We further validated the four top differentially expressed lncRNAs, GM18840, Meg3, Dnm3os, and Pvt1, using qRT-PCR. As shown in Figure 1F, the relative gene expression levels of GM18840, Meg3, Dnm3os, Pvt1, and, especially, Gm18840 were significantly upregulated in cardiac tissues with IRI treatment.

### Validation of Identified lncRNAs Using a Simulated IRI Model in HL-1 Cells

**FIGURE 2:**
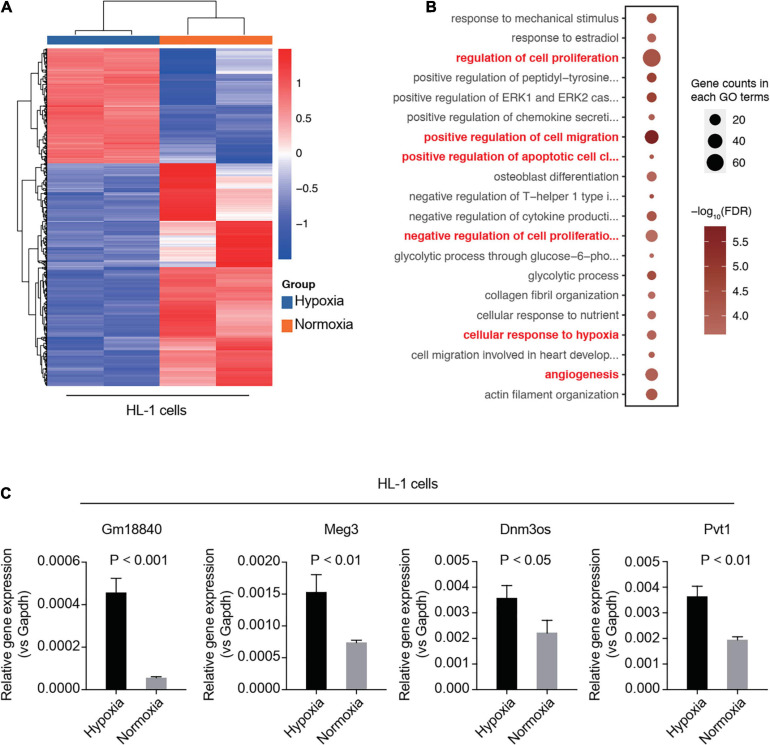
Validation of identified lncRNAs using a simulated ischemia/reperfusion model in HL-1 cells. **(A,B)** RNA-seq analysis in an *in vitro* model of simulated ischemia. HL-1 cells were cultured under normoxia or hypoxia. Differentially expressed genes between normoxia and hypoxia in HL-1 cells are shown **(A)**. Gene ontology analysis was performed using the differentially expressed genes **(B)**. **(C)** Validation of the differentially expressed lncRNAs by qRT-PCR in the murine cardiac muscle cell line HL-1 upon hypoxia treatment. HL-1 cells were cultured with 20% oxygen (normoxia) or 1% oxygen (hypoxia) for 24 h. The expression of these genes was normalized to GAPDH.

To validate the function of GM18840, Meg3, Dnm3os, and Pvt1 in MIRI, we carried out RNA-seq analysis in an *in vitro* model of simulated ischemia. We investigated the DEGs in HL-1 cells under normoxia or hypoxia. Many genes showed an overlap between the mouse model and the cell line model (Supplementary Figure 2). The heatmap of DEGs between normoxia and hypoxia in HL-1 cells is shown in Figure 2A and the differentially expressed lncRNAs in the heart tissue of the mice with/without MIRI are shown in Supplementary Table 4. GO analysis was performed based on the above DEGs. Similar to those observed in the mouse model, genes related to the cellular responses to hypoxia, angiogenesis, negative regulation of cell proliferation, and positive regulation of cell migration were enriched (Figure 2B). In addition, we detected the relative expression levels of Gm18840, Meg3, Dnm3os, and Pvt1 in the murine cardiac muscle cell line HL-1 upon hypoxia treatment by qRT-PCR. The results revealed that these four differentially expressed lncRNAs, especially Gm18840, were also significantly upregulated in HL-1 cells upon hypoxia treatment (Figure 2C).

**FIGURE 3:**
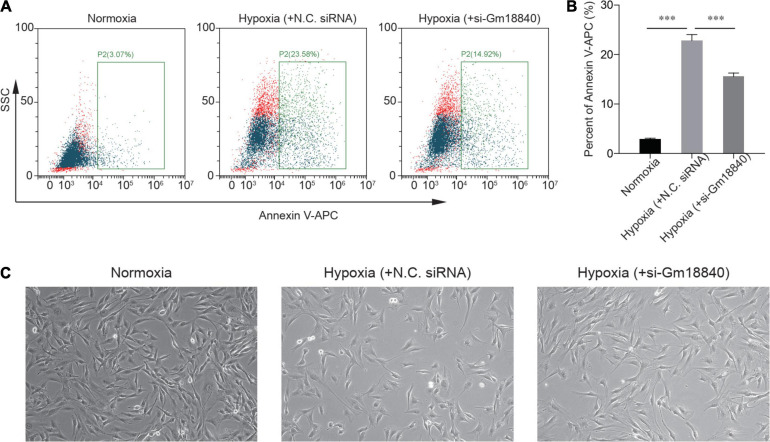
Knockdown of Gm18840 reduced hypoxia-induced apoptosis in cardiac muscle cells. **(A,B)** Knockdown of Gm18840 decreased hypoxia-resultant apoptosis in HL-1 cells. Representative flow cytometry **(A)** and statistical analysis **(B)** are shown. HL-1 cells were treated with siRNA targeting Gm18840 before hypoxia experiments. For the hypoxia experiments, HL-1 cells were cultured in 20% oxygen (normoxia) or 1% oxygen (hypoxia) for 24 h. The cells were harvested 24 h after exposure to hypoxia or normoxia culture. **(C)** Morphology of HL-1 cells under normoxia, hypoxia with negative control (NC) siRNA, or hypoxia with siRNA targeting Gm18840. ***p < 0.001.

We further explored the function of Gm18840 in the regulation of myocardial ischemia–reperfusion injury. We found that the knockdown of Gm18840 significantly decreased hypoxia-resultant apoptosis in HL-1 cells according to flow cytometry experiments (Figures 3A,B). The morphology results showed that the cell density and the degree of damage of the Si-Gm18840 group were significantly improved compared with those of the hypoxia group. The morphology of HL-1 cells under normoxia, hypoxia with negative control (NC) siRNA, or hypoxia with siRNA targeting Gm18840 was consistent with the flow cytometry results (Figure 3C). Furthermore, Gm18840 is a novel non-coding RNA that has never been reported; hence, we chose Gm18840 as a target in the following experiment. The common deregulated genes in the different RNA-seq analyses are shown in Supplementary Figure 2.

### Identification and Characterization of Gm18840

**FIGURE 4:**
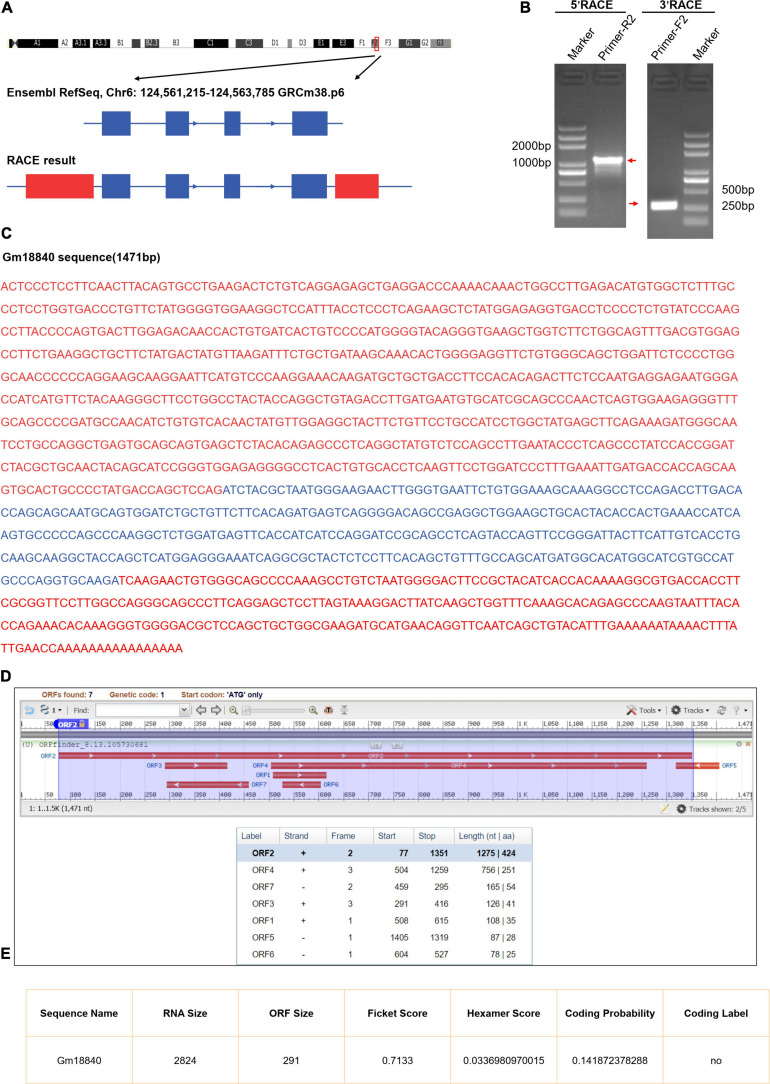
Identification and characterization of Gm18840. **(A)** Schematic diagram of Gm18840 from Ensembl RefSeq and RACE. Blue boxes: exons annotated by NCBI RefSeq. Red boxes: additional sequences and exons determined by RACE. Blue lines: introns. Arrows on blue lines indicate transcriptional directions. **(B)** Gel electrophoresis of nested PCR products from 5′-RACE and 3′-RACE. Red arrows indicate product bands. **(C)** The full-length sequence of the Gm18840 transcript. Blue: reference sequence from NCBI RefSeq. Red: sequence from RACE. **(D,E)** ORF finder software **(D)** and CPAT software. **(E)** Prediction of the protein-coding potential of Gm18840.

From the UCSC Genome Browser,^1^ we found that Gm18840 is located on mouse chromosome 6F2 and composed of four exons annotated by Ensembl RefSeq (exon 1, 146 bp; exon 2, 122 bp; exon 3, 159 bp; exon 4, 185 bp) (Figure 4A). To identify the full-length Gm18840 transcript in HL-1 cells, a RACE assay was performed, and the PCR products of 5′- and 3′-RACE are shown in Figure 4B. Based on the Sanger sequencing results, the full-length Gm18840 transcript was found to be 1,471 nt, with an additional 1,119 bp at the 5′ end of exon 1 and an additional 320 bp at the 3′ end of exon 4 (Figures 4A,C). However, the ORF finder from NCBI^2^ failed to predict a protein from the Gm18840 sequence, as determined by RACE (Figure 4D). The CPAT database^3^ also showed that Gm18840 had limited protein-coding potential (coding probability = 0.141872378288) (Figure 4E).

### Interaction of Gm18840 With Histone H2B in HL-1 Cells

**FIGURE 5:**
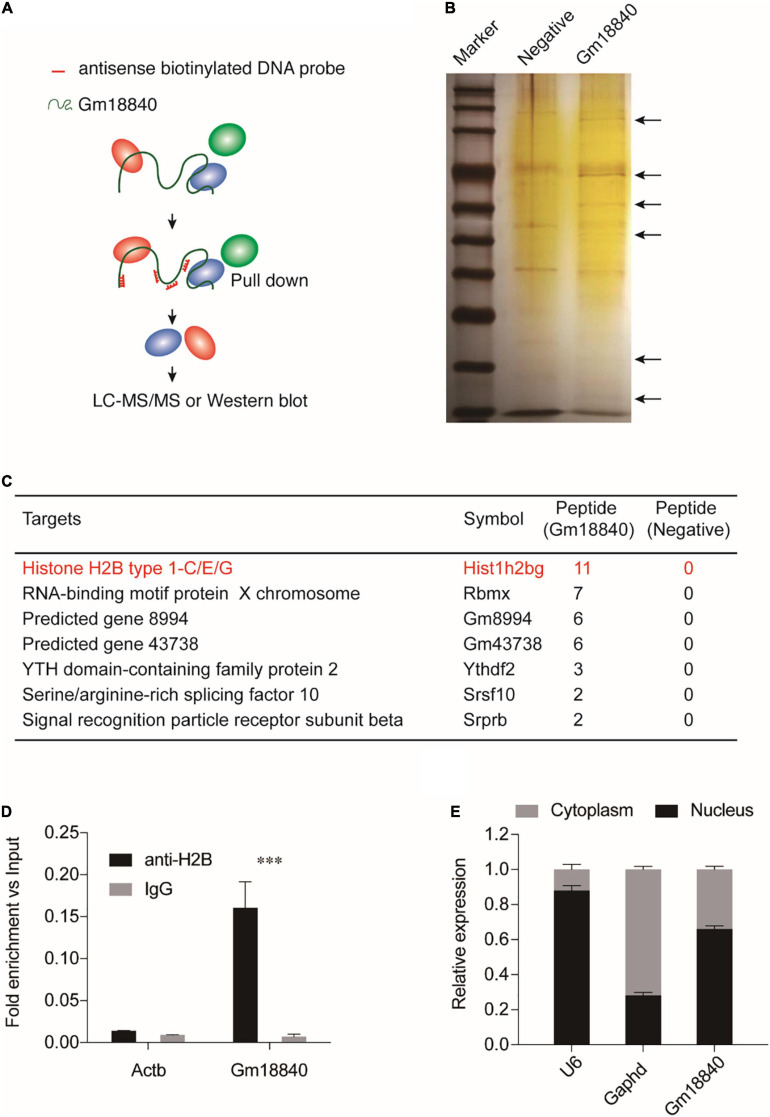
Gm18840 interacted with histone H2B and was located in the nucleus and cytoplasm. **(A)** Schematic illustration of RNA pulldown assays. Biotinylated DNA against the Gm18840 lncRNA was used for RNA pulldown analysis. HL-1 cells were fixed with formaldehyde and sonicated. The Gm18840 lncRNA was hybridized with DNA probes, and the interacting protein of Gm18840 was captured. Primers against Gapdh were used as the negative control. **(B)** Silver staining showing the proteins uniquely bound by Gm18840 in HL-1 cells. **(C)** LC-MS/MS analysis of the Gm18840-interacting proteins. **(D)** RIP-qPCR analysis of the direct binding between H2B protein and the Gm18840 lncRNAs in HL-1 cells. **(E)** Distribution of Gm18840 in the cytoplasm and the nucleus. RNA was extracted from the cytoplasm and the nucleus, and the expression of U6 (mainly distributed in the nucleus), Gapdh (mainly distributed in the cytoplasm), and Gm18840 was detected. ***p < 0.001.

In the next step, RNA pulldown assays were performed coupled with LC-MS/MS to investigate the interaction of Gm18840 in HL-1 cells (Supplementary Table 5). As shown in Figure 5A, the Gm18840 lncRNA was hybridized with biotinylated DNA probes, and the interacting protein of Gm18840 was captured. Silver staining results for the proteins pulled down by Gm18840 indicated that Gm18840 can specifically bind to certain proteins (Figure 5B). Moreover, seven proteins that could directly interact with Gm18840 with high confidence were revealed by the LC-MS/MS results (Figure 5C). Among them, histone H2B was the most reliable. Furthermore, RIP-qPCR analysis suggested that there was direct binding between the H2B protein and the Gm18840 lncRNA in HL-1 cells (Figure 5D). Notably, the identified targets were core histone proteins, suggesting the possible transcriptional regulatory function of Gm18840. In addition, we determined the distribution of Gm18840 lncRNA in the cytoplasm and the nucleus. We found that Gm18840 was mainly expressed in the nucleus, further suggesting its potential function in transcriptional regulation (Figure 5E).

### Gm18840 Is Involved in Transcriptional Regulation Through Direct Binding to Regulatory Regions in Cardiac Muscle Cells

**FIGURE 6:**
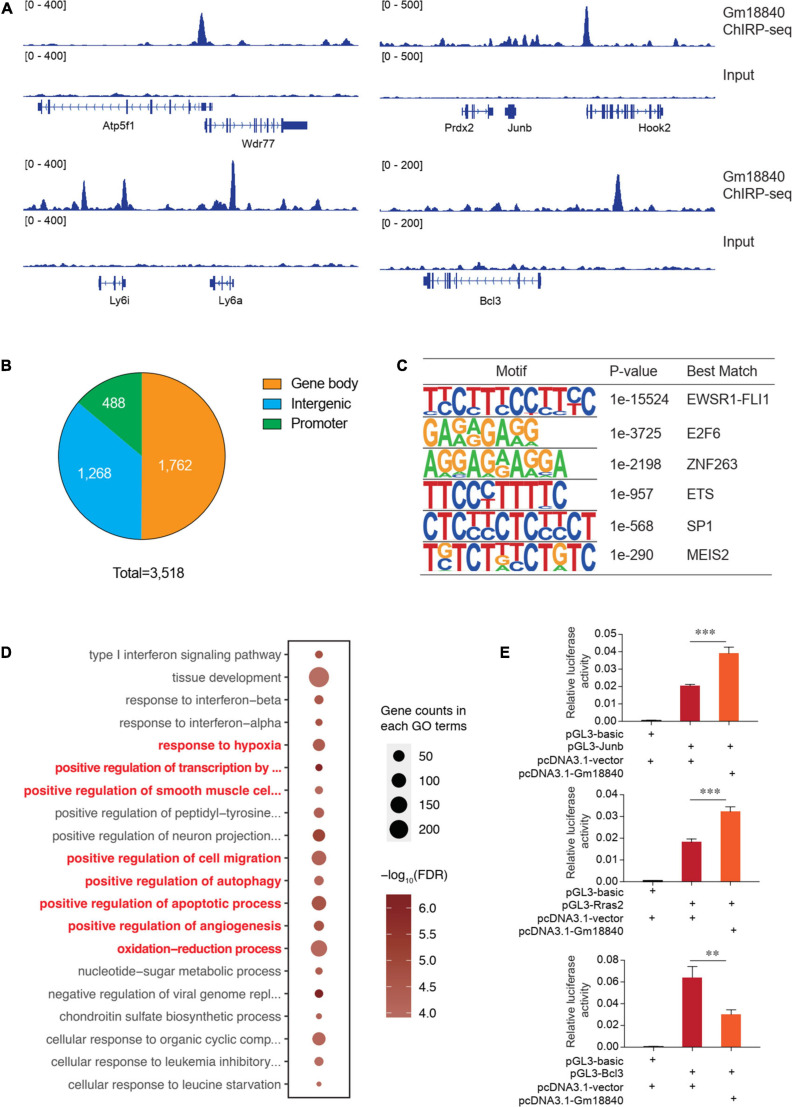
Gm18840 is involved in transcriptional regulation in cardiac muscle cells. **(A)** Genome-browser screen showing the binding of Gm18840 in the regulatory regions of representative genes. ChIRP-seq experiments were conducted to determine the genome-wide binding of Gm18840 to chromatin. Representative binding among the regulatory regions of Wdr77, Junb, Ly6a, and Bcl3 is shown. **(B)** Distribution of Gm18840-bound regions. The numbers of Gm18840-bound peaks in the gene body, promoter, and intergenic regions are shown. **(C)** Motif analysis of Gm18840-bound regions. The motif analysis was performed with the Homer suite. The EWSR1-FLI1, E2F6, ZNF263, ETS, SP1, and MEIS2 motifs were highly enriched, implying the coregulation of Gm18840 with these factors. **(D)** Gene ontology analysis of genes associated with Gm18840-bound peaks. **(E)** Gm18840 directly regulated the transcriptional activity of its targets. Luciferase reporter assays were performed in HL-1 cells. The representative regions bound by Gm18840, including Junb, Rras2, and Bcl3, were cloned into the pGL3 plasmid. The luciferase constructs were cotransfected with the pcDNA3.1 vector or pcDNA3.1-Gm18840 plasmids. The firefly luciferase activity was normalized to Renilla luciferase activity. **p < 0.01 and ***p < 0.001.

To research the direct targets of transcriptional regulation by Gm18840, ChIRP-seq experiments were conducted using biotinylated antisense DNA probe sets. A genome-browser screen showed the binding of Gm18840 in the promoters of representative genes among the regulatory regions of *Atp5f1, Wdr77, Prdx2, Junb, Hook2, Ly6i, Ly6a*, and *Bcl3* (Figure 6A). In addition, we identified 3,518 Gm18840 targets that directly bind to regions on chromatin (Supplementary Table 6). Of these regions, 13.9% were promoters, whereas others were regions on the gene (Figure 6B). Furthermore, motif enrichment analysis of these regions was performed, and the motifs EWSR1-FLI1, E2F6, ZNF263, ETS, SP1, and MEIS2 were significantly enriched (Figure 6C), implying a coregulation of Gm18840 with these factors. As shown in Figure 6D, the results of GO analysis of genes associated with Gm18840-bound peaks revealed that Gm18840 plays an important role in the response to hypoxia, positive regulation of cell migration, positive regulation of autophagy, positive regulation of apoptotic processes, and positive regulation of angiogenesis, indicating an essential role for Gm18840 in MIRI. Luciferase reporter assays performed in HL-1 cells showed that Gm18840 directly regulated the transcriptional activity of its targets, such as Junb, Rras2, and Bcl3 (Figure 6E and Supplementary Table 7), indicating an important function of Gm18840 in gene regulation.

**FIGURE 7:**
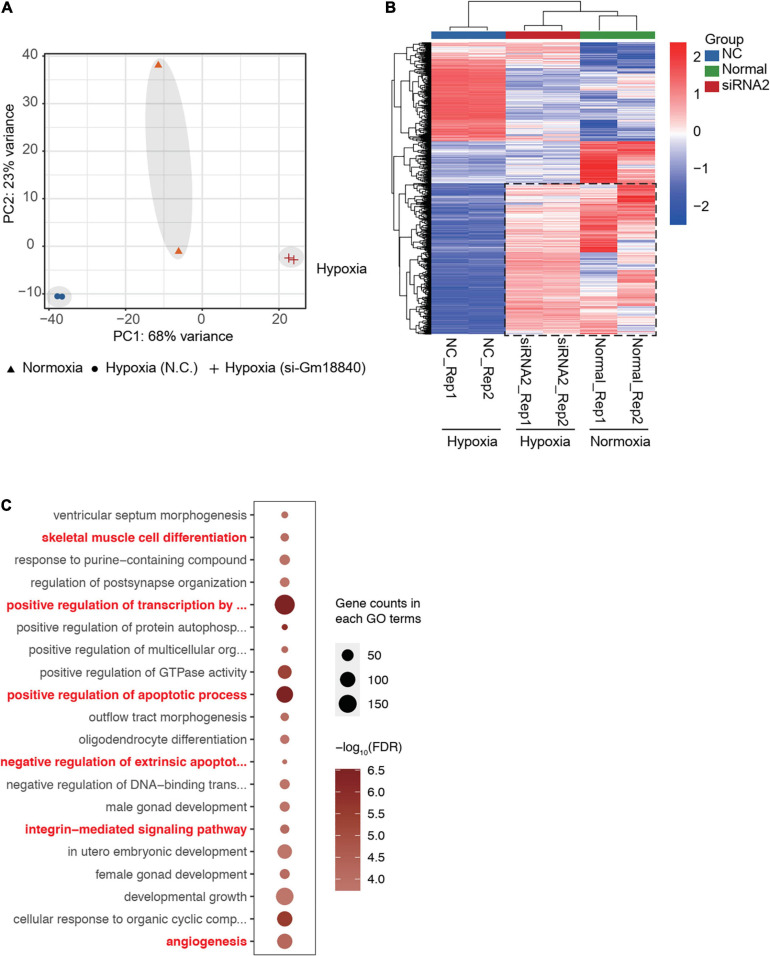
Gm18840 partially restored the transcriptional pattern under hypoxia to that under normoxia. **(A)** PCA of the transcriptome of HL-1 cells under normoxia and HL-1 cells transfected with negative control siRNA or siRNA targeting Gm18840 under hypoxia. The transcriptomes of HL-1 cells under normoxia and HL-1 cells with Gm18840 knockdown under hypoxia were similar. **(B)** Knockdown of Gm18840 under hypoxia partially restored gene expression to that under normoxia. Heatmap showing the expression of genes differentially expressed in normoxia (Normal group) compared to hypoxia (NC group). Relative expression of these genes in HL-1 cells under normoxia (Normal group) and HL-1 cells transfected with negative control siRNA or siRNA targeting Gm18840 under hypoxia (SiRNA group) are shown. **(C)** Gene ontology analysis of genes directly regulated by Gm18840. Genes directly regulated by Gm18840 were defined by ChIRP-seq binding and differentially expressed upon Gm18840 knockdown.

To further understand the effects of Gm18840 on transcriptional regulation, we performed RNA-seq experiments in HL-1 cells under normoxia and HL-1 cells with Gm18840 knockdown. The PCA results showed that the transcriptomes of HL-1 cells under normoxia and HL-1 cells with Gm18840 knockdown under hypoxia were similar (Figure 7A). In addition, as shown in Figure 7B and Supplementary Table 8, a heatmap displaying the expression of genes differentially expressed in normoxia compared to hypoxia indicated that the knockdown of Gm18840 under hypoxia partially restored gene expression to that under normoxia. With the knockdown of Gm18840 under hypoxia, the relative expression of Junb, Rras2, and Bcl3 increased, indicating that Gm18840 influenced the activity of the promoters of Junb and Bcl3 (Supplementary Figure 3). Gm18840 directly regulated genes that were defined by ChIRP-seq binding and differentially expressed upon the Gm18840 knockdown. As shown in Figure 7C, the enriched GO items of Gm18840 directly regulated genes similar to those in hypoxia vs normoxia as identified in Figures 1D, 2B, 6D. These results suggested that Gm18840 partially restored the transcriptional pattern under hypoxia to that under normoxia.

## Discussion

MI is one of the major causes of hospitalization and death worldwide (Zidan et al., 2016; Chen et al., 2018). Therefore, studying molecular mechanisms and discovering impactful therapeutic targets are important for the treatment of cardiac ischemia-related disease. LncRNAs belong to the largest category of RNAs and are closely related to various human diseases (Liao et al., 2011; Huo et al., 2017). They are key regulators of various biological processes (Bhan et al., 2017; Kondo et al., 2017).

Recently, various studies have emphasized that multiple lncRNAs play important roles in the occurrence and development of cardiac ischemia-related disease (Jiang and Ning, 2015; Fa et al., 2020; Huang, 2020; Pierce and Feinberg, 2020). Chen et al. (2018) demonstrated that the expression of lncRNA UCA1 is correlated with I/R-induced cardiac injury in rats. Zhu and Zhao (2019) showed that the expression of lncRNA AK139128 in MIRI tissues and myocardial cells is upregulated, and MIR can be alleviated with AK139128 interference by inhibiting myocardial cell apoptosis. Hence, I/R injury might be improved by modulating the expression levels of lncRNAs. In this study, we first identified differentially expressed lncRNAs by performing RNA-seq analysis in the heart tissue of the mice with or without MIRI. Then, validation by qRT-PCR in cardiac tissues and RNA-seq and qRT-PCR detection in I/R HL-1 cells revealed the involvement of Gm18840, which was selected for further investigation in MI.

Apoptosis is a cell death mechanism induced by various stress conditions, such as oxidative stress and endoplasmic reticulum stress. It has been widely demonstrated that cardiac apoptosis is dramatically enhanced following myocardial ischemic injury (Frankenreiter et al., 2017; Tanimoto et al., 2017; Yao et al., 2017). Many studies have shown that lncRNAs, such as H19 (Li et al., 2019), MALAT1 (Guo X. et al., 2019; Sun et al., 2019), and MEG3 (Wu et al., 2018), play multiple functions during myocardial ischemic injury. No previous studies have demonstrated that Gm18840 is related to MIRI. We reported that the regulatory role of lncRNA Gm18840 in cardiomyocyte apoptosis after hypoxia/reperfusion injury is meaningful. We found that Gm18840 could inhibit apoptosis in I/R HL-1 cells. Interestingly, we did not observe a significant increase in Gm18840 in the RNA-Seq results based on HL-1 cells but verified that Gm18840 was significantly upregulated *in vivo* and *in vitro* via qRT-PCR. The reason for this may be that there are more cell types in tissues, and the effect is more complicated; considering that the sample size in the RNA-seq experiment was relatively small, false-positive results were prone to occur. The results above provide an impetus to further study the function and mechanism of Gm18840 in MIRI.

The structure and localization of lncRNAs in cells are closely related to their functions and mechanisms (Chen, 2016; MacDonald and Mann, 2020). Gm18840 showed limited protein-coding potential. Nucleocytoplasmic separation showed that Gm18840 was mainly located in the nucleus. This may suggest that Gm18840 is involved in transcriptional regulation in I/R HL-1 cells. At the transcriptional level, lncRNA bridges DNA and protein by binding to chromatin and serving as a scaffold for the modification of protein complexes. Such a mechanism can bridge promoters to enhancers or enhancer-like non-coding genes by regulating chromatin looping, as well as conferring specificity upon histone modifying complexes by directing them to specific loci (Dykes and Emanueli, 2017).

Recent studies on the function of lncRNAs have revealed the direct histone binding and transcriptional regulation functions of lncRNAs. For example, MALAT1, which is an lncRNA, directly interacts with DBC1 and P53 and regulates the acetylation of the P53 protein (Chen et al., 2017). Here, we demonstrated that there was direct binding between the H2B protein and Gm18840 lncRNAs in HL-1 cells. In addition, the results showed that Gm18840 was mainly expressed in the nucleus, indicating that Gm18840 directly interacts with the nucleosome-related protein histone H2B and is involved in transcriptional regulation in HL-1 cells. Posttranslational regulation of H2B has been reported to be essential for myocardial development (Robson et al., 2019). Transcriptional regulation plays a vital role in almost all biological processes in myocardial development and disease. A large amount of evidence has shown that lncRNAs interact with histones to play a role in the development of disease (Jain et al., 2016; Sun et al., 2016). To further research the direct targets of transcriptional regulation by Gm18840, ChIRP-seq and luciferase reporter assays were conducted. Our ChIRP-seq and RNA-seq results suggested that Gm18840, as a transcriptional modulator, directly binds to the chromatin regulation regions of many essential genes, such as Atp5f1, Junb, Ly6a, and Bcl3. Gm18840 regulated the promoter/enhancer activity of these genes, including Junb, Rras2, and Bcl3. Motif enrichment analysis showed that Gm18840 bound to the region enriched for the EWSR1-FLI1, E2F6, and ZNF263 motifs, indicating that Gm18840 might regulate the transcriptional activity of these transcription factors and contribute to myocardial ischemia–reperfusion injury. Nevertheless, the effects of the Gm18840-H2B interaction must be investigated in the future. Gm18840 might interact with H2B and directly bind to the transcriptional regulatory regions of the genes essential for the promotion of apoptosis, such as Junb, Rras2, and Bcl3. Junb is a gene belonging to the AP-1 transcriptional complex that is suppressed and has been reported to suppress cell apoptosis in heart failure (Yan et al., 2018). Sabatasso et al. (2018) confirmed that JunB is an early, sensitive marker for myocardial ischemia. Schmidt et al. (2007) indicated that JunB is a target gene of hypoxia-induced signaling via NF-kappaB. Bcl3 is a proto-oncogene that belongs to the IκB family, and it has been reported that BCL3 can exert an anti-apoptotic effect. Lee et al. (2015) revealed that Bcl3 was upregulated under hypoxic conditions, resulting in the prevention of apoptosis induced by ischemic stress both *in vitro* and *in vivo*. We found that low expression of Gm18840 upregulated the expression of Junb and Bcl3, indicating that Gm18840 directly inhibits cell apoptosis in I/R HL-1 cells.

In conclusion, we profiled MIRI-related protein-coding and non-coding RNAs and identified an essential regulator, Gm18840, for MIRI. We demonstrated that Gm18840 was upregulated in MIRI tissues and hypoxic HL-1 cells. These results suggest that Gm18840 might promote myocardial cell apoptosis in MIRI and regulate cardiomyocyte apoptosis after hypoxia/reperfusion injury. However, the molecular mechanism of Gm18840 is still unclear. The role of the Gm18840-mediated regulatory network and its association with other apoptosis factors should be explored in the future.

## Data Availability Statement

The datasets presented in this study can be found in online repositories. The names of the repository/repositories and accession number(s) can be found below: SRA Database (PRJNA669226).

## Ethics Statement

The animal study was reviewed and approved by Animal Care and Use Committee of Affiliated Liutie Central Hospital & Clinical Medical College of Guangxi Medical University.

## Author Contributions

CL, XK, and SX designed the research. CL and XK carried out the analyses. YH and MD performed the molecular cell experiments. JZ, JC, and RY performed the visualization. SX and XK wrote the manuscript. All authors reviewed and approved the final manuscript.

## Conflict of Interest

The authors declare that the research was conducted in the absence of any commercial or financial relationships that could be construed as a potential conflict of interest.

## Footnotes

1 http://genome.ucsc.edu

2 https://www.ncbi.nlm.nih.gov/orffinder/

3 http://lilab.research.bcm.edu/cpat/

## References

[B1] Anders S. Pyl P. T. Huber W. (2015). HTSeq–a Python framework to work with high-throughput sequencing data. *Bioinformatics* 31 166–169. 10.1093/bioinformatics/btu638 25260700 PMC4287950

[B2] Bailey T. L. Johnson J. Grant C. E. Noble W. S. (2015). The MEME suite. *Nucleic Acids Res.* 43 W39–W49. 25953851 10.1093/nar/gkv416 PMC4489269

[B3] Bhan A. Soleimani M. Mandal S. (2017). Long noncoding RNA and cancer: a new paradigm. *Cancer Res.* 77 3965–3981. 10.1158/0008-5472.can-16-2634 28701486 PMC8330958

[B4] Chen L. L. (2016). Linking long noncoding RNA localization and function. *Trends Biochem. Sci.* 41 761–772. 10.1016/j.tibs.2016.07.003 27499234

[B5] Chen R. Liu Y. Zhuang H. Yang B. Hei K. Xiao M. (2017). Quantitative proteomics reveals that long non-coding RNA MALAT1 interacts with DBC1 to regulate p53 acetylation. *Nucleic Acids Res.* 45 9947–9959. 10.1093/nar/gkx600 28973437 PMC5622371

[B6] Chen Z. Liu R. Niu Q. Wang H. Yang Z. Bao Y. (2018). Morphine postconditioning alleviates autophage in ischemia-reperfusion induced cardiac injury through up-regulating lncRNA UCA1. *Biomed. Pharmacother.* 108 1357–1364. 10.1016/j.biopha.2018.09.119 30372838

[B7] Chu C. Quinn J. Chang H. Y. (2012). Chromatin isolation by RNA purification (ChIRP). *J. Vis. Exp.* 61 3912. 10.3791/3912 PMC3460573 22472705

[B8] Dobin A. Davis C. A. Schlesinger F. Drenkow J. Zaleski C. Jha S. (2013). STAR: ultrafast universal RNA-seq aligner. *Bioinformatics* 29 15–21. 10.1093/bioinformatics/bts635 23104886 PMC3530905

[B9] Dykes I. M. Emanueli C. (2017). Transcriptional and post-transcriptional gene regulation by long non-coding RNA. *Genomics Proteomics Bioinformatics* 15 177–186. 10.1016/j.gpb.2016.12.005 28529100 PMC5487525

[B10] Fa H. Chang W. G. Zhang X. J. Xiao D. D. Wang J. X. (2020). Noncoding RNAs in doxorubicin-induced cardiotoxicity and their potential as biomarkers and therapeutic targets. *Acta Pharmacol. Sin.* 42 499–507. 10.1038/s41401-020-0471-x 32694762 PMC8114921

[B11] Fan Y. Z. Huang H. Wang S. Tan G. J. Zhang Q. Z. (2019). Effect of lncRNA MALAT1 on rats with myocardial infarction through regulating ERK/MAPK signaling pathway. *Eur. Rev. Med. Pharmacol. Sci.* 23 9041–9049. 31696494 10.26355/eurrev_201910_19306

[B12] Frankenreiter S. Bednarczyk P. Kniess A. Bork N. I. Straubinger J. Koprowski P. (2017). cGMP-elevating compounds and ischemic conditioning provide cardioprotection against ischemia and reperfusion injury via cardiomyocyte-specific BK channels. *Circulation* 136 2337–2355. 10.1161/circulationaha.117.028723 29051185

[B13] Guo G. L. Sun L. Q. Sun M. H. Xu H. M. (2019). LncRNA SLC8A1-AS1 protects against myocardial damage through activation of cGMP-PKG signaling pathway by inhibiting SLC8A1 in mice models of myocardial infarction. *J. Cell. Physiol.* 234 9019–9032. 10.1002/jcp.27574 30378115

[B14] Guo X. Wu X. Han Y. Tian E. Cheng J. (2019). LncRNA MALAT1 protects cardiomyocytes from isoproterenol-induced apoptosis through sponging miR-558 to enhance ULK1-mediated protective autophagy. *J. Cell. Physiol.* 234 10842–10854. 10.1002/jcp.27925 30536615

[B15] Hausenloy D. J. Erik Bøtker H. Condorelli G. Ferdinandy P. Garcia-Dorado D. Heusch G. (2013). Translating cardioprotection for patient benefit: position paper from the working group of cellular biology of the heart of the European Society of Cardiology. *Cardiovasc. Res.* 98 7–27. 10.1093/cvr/cvt004 23334258

[B16] Huang Y. (2020). Exosomal lncRNAs from mesenchymal stem cells as the novel modulators to cardiovascular disease. *Stem Cell Res. Ther.* 11:315. 32703265 10.1186/s13287-020-01812-6 PMC7376709

[B17] Huang da W. Sherman B. T. Lempicki R. A. (2009). Bioinformatics enrichment tools: paths toward the comprehensive functional analysis of large gene lists. *Nucleic Acids Res.* 37 1–13. 10.1093/nar/gkn923 19033363 PMC2615629

[B18] Huo X. Han S. Wu G. Latchoumanin O. Zhou G. Hebbard L. (2017). Dysregulated long noncoding RNAs (lncRNAs) in hepatocellular carcinoma: implications for tumorigenesis, disease progression, and liver cancer stem cells. *Mol. Cancer* 16:165. 29061150 10.1186/s12943-017-0734-4 PMC5651571

[B19] Jain A. K. Xi Y. McCarthy R. Allton K. Akdemir K. C. Akdemir K. C. (2016). LncPRESS1 is a p53-regulated LncRNA that safeguards pluripotency by disrupting SIRT6-mediated de-acetylation of histone H3K56. *Mol. Cell* 64 967–981. 10.1016/j.molcel.2016.10.039 27912097 PMC5137794

[B20] Jiang X. Ning Q. (2015). The emerging roles of long noncoding RNAs in common cardiovascular diseases. *Hypertens. Res.* 38 375–379. 10.1038/hr.2015.26 25762413

[B21] Jiao L. Li M. Shao Y. Zhang Y. Gong M. Yang X. (2019). lncRNA-ZFAS1 induces mitochondria-mediated apoptosis by causing cytosolic Ca(2+) overload in myocardial infarction mice model. *Cell Death Dis.* 10:942. 31819041 10.1038/s41419-019-2136-6 PMC6901475

[B22] Kenneweg F. Bang C. Xiao K. Boulanger C. M. Loyer X. Mazlan S. (2019). Long noncoding RNA-enriched vesicles secreted by hypoxic cardiomyocytes drive cardiac fibrosis. *Mol. Ther. Nucleic Acids* 18 363–374. 10.1016/j.omtn.2019.09.003 31634682 PMC6807307

[B23] Kondo Y. Shinjo K. Katsushima K. (2017). Long non-coding RNAs as an epigenetic regulator in human cancers. *Cancer Sci.* 108 1927–1933. 10.1111/cas.13342 28776911 PMC5623749

[B24] Langmead B. Salzberg S. L. (2012). Fast gapped-read alignment with Bowtie 2. *Nat. Methods* 9 357–359. 10.1038/nmeth.1923 22388286 PMC3322381

[B25] Lee S. Lee J. H. Han Y. S. Ryu J. M. Yoon Y. M. Han H. J. (2015). Hypoxia accelerates vascular repair of endothelial colony-forming cells on ischemic injury via STAT3-BCL3 axis. *Stem Cell Res. Ther.* 6:139. 26219963 10.1186/s13287-015-0128-8 PMC4522108

[B26] Li X. Luo S. Zhang J. Yuan Y. Jiang W. Zhu H. (2019). lncRNA H19 alleviated myocardial I/RI via suppressing miR-877-3p/Bcl-2-mediated mitochondrial apoptosis. *Mol. Ther. Nucleic Acids* 17 297–309. 10.1016/j.omtn.2019.05.031 31284127 PMC6612907

[B27] Li Z. Sun X. (2020). Non-coding RNAs operate in the crosstalk between cancer metabolic reprogramming and metastasis. *Front. Oncol.* 10:810. 32547948 10.3389/fonc.2020.00810 PMC7273922

[B28] Liao Q. Liu C. Yuan X. Kang S. Miao R. Xiao H. (2011). Large-scale prediction of long non-coding RNA functions in a coding-non-coding gene co-expression network. *Nucleic Acids Res.* 39 3864–3878. 10.1093/nar/gkq1348 21247874 PMC3089475

[B29] Lopez A. D. Mathers C. D. Ezzati M. Jamison D. T. Murray C. J. (2006). Global and regional burden of disease and risk factors, 2001: systematic analysis of population health data. *Lancet* 367 1747–1757. 10.1016/s0140-6736(06)68770-9 16731270

[B30] Love M. I. Huber W. Anders S. (2014). Moderated estimation of fold change and dispersion for RNA-seq data with DESeq2. *Genome Biol.* 15:550. 25516281 10.1186/s13059-014-0550-8 PMC4302049

[B31] Lu J. Xu F. Q. Guo J. J. Lin P. L. Meng Z. Hu L. G. (2019). Long noncoding RNA GAS5 attenuates cardiac fibroblast proliferation in atrial fibrillation via repressing ALK5. *Eur. Rev. Med. Pharmacol. Sci.* 23 7605–7610. 31539152 10.26355/eurrev_201909_18883

[B32] MacDonald W. A. Mann M. R. W. (2020). Long noncoding RNA functionality in imprinted domain regulation. *PLoS Genet.* 16:e1008930. 10.1371/journal.pgen.1008930 32760061 PMC7410213

[B33] Matyas C. Németh B. T. Oláh A. Török M. Ruppert M. Kellermayer D. (2017). Prevention of the development of heart failure with preserved ejection fraction by the phosphodiesterase-5A inhibitor vardenafil in rats with type 2 diabetes. *Eur. J. Heart Fail.* 19 326–336. 10.1002/ejhf.711 27995696 PMC5347963

[B34] Ong S. B. Hernández-Reséndiz S. Crespo-Avilan G. E. Mukhametshina R. T. Kwek X. Y. Cabrera-Fuentes H. A. (2018). Inflammation following acute myocardial infarction: multiple players, dynamic roles, and novel therapeutic opportunities. *Pharmacol. Ther.* 186 73–87. 10.1016/j.pharmthera.2018.01.001 29330085 PMC5981007

[B35] Pierce J. Feinberg M. (2020). Long noncoding RNAs in atherosclerosis and vascular injury: pathobiology, biomarkers, and targets for therapy. *Arterioscler. Thromb. Vasc. Biol.* 40 2002–2017. 10.1161/atvbaha.120.314222 32698685 PMC7484026

[B36] Reed G. W. Rossi J. E. Cannon C. P. (2017). Acute myocardial infarction. *Lancet* 389 197–210. 27502078 10.1016/S0140-6736(16)30677-8

[B37] Robson A. Makova S. Z. Barish S. Zaidi S. Mehta S. Drozd J. (2019). Histone H2B monoubiquitination regulates heart development via epigenetic control of cilia motility. *Proc. Natl. Acad. Sci. U.S.A.* 116 14049–14054. 10.1073/pnas.1808341116 31235600 PMC6628794

[B38] Sabatasso S. Moretti M. Mangin P. Fracasso T. (2018). Early markers of myocardial ischemia: from the experimental model to forensic pathology cases of sudden cardiac death. *Int. J. Legal Med.* 132 197–203. 10.1007/s00414-017-1605-7 28497398

[B39] Sayad A. Mirzajani S. Gholami L. Razzaghi P. Ghafouri-Fard S. Taheri M. (2020). Emerging role of long non-coding RNAs in the pathogenesis of periodontitis. *Biomed. Pharmacother.* 129:110362. 10.1016/j.biopha.2020.110362 32563981

[B40] Schmidt D. Textor B. Pein O. T. Licht A. H. Andrecht S. Sator-Schmitt M. (2007). Critical role for NF-kappaB-induced JunB in VEGF regulation and tumor angiogenesis. *EMBO J.* 26 710–719. 10.1038/sj.emboj.7601539 17255940 PMC1794395

[B41] Sun T. Cheng Y. T. Yan L. X. Krittanawong C. Qian W. Zhang H. J. (2019). LncRNA MALAT1 knockdown alleviates myocardial apoptosis in rats with myocardial ischemia-reperfusion through activating PI3K/AKT signaling pathway. *Eur. Rev. Med. Pharmacol. Sci.* 23 10523–10531. 31841208 10.26355/eurrev_201912_19693

[B42] Sun T. T. He J. Liang Q. Ren L. L. Yan T. T. Yu T. C. (2016). LncRNA GClnc1 promotes gastric carcinogenesis and may act as a modular scaffold of WDR5 and KAT2A complexes to specify the histone modification pattern. *Cancer Discov.* 6 784–801. 10.1158/2159-8290.cd-15-0921 27147598

[B43] Tanimoto T. Parseghian M. H. Nakahara T. Kawai H. Narula N. Kim D. (2017). Cardioprotective effects of hsp72administration on ischemia-reperfusion injury. *J. Am. Coll. Cardiol.* 70 1479–1492. 28911512 10.1016/j.jacc.2017.07.762 PMC5659834

[B44] Viereck J. Kumarswamy R. Foinquinos A. Xiao K. Avramopoulos P. Kunz M. (2016). Long noncoding RNA Chast promotes cardiac remodeling. *Sci. Transl. Med.* 8:326ra22. 10.1126/scitranslmed.aaf1475 26888430

[B45] Wang K. Liu C. Y. Zhou L. Y. Wang J. X. Wang M. Zhao B. (2015). APF lncRNA regulates autophagy and myocardial infarction by targeting miR-188-3p. *Nat. Commun.* 6:779. 10.1038/ncomms7779 25858075

[B46] Wu H. Zhao Z. A. Liu J. Hao K. Yu Y. Han X. (2018). Long noncoding RNA Meg3 regulates cardiomyocyte apoptosis in myocardial infarction. *Gene Ther.* 25 511–523. 10.1038/s41434-018-0045-4 30287867

[B47] Yan M. Yang S. Meng F. Zhao Z. Tian Z. Yang P. (2018). MicroRNA 199a-5p induces apoptosis by targeting JunB. *Sci. Rep.* 8:6699. 29703907 10.1038/s41598-018-24932-9 PMC5923206

[B48] Yang X. Tao L. Zhu J. Zhang S. (2019). Long noncoding RNA FTX reduces hypertrophy of neonatal mouse cardiac myocytes and regulates the PTEN/PI3K/Akt signaling pathway by sponging MicroRNA-22. *Med. Sci. Monit.* 25 9609–9617. 10.12659/msm.919654 31840653 PMC6929539

[B49] Yao Y. Lu Q. Hu Z. Yu Y. Chen Q. Wang Q. K. (2017). A non-canonical pathway regulates ER stress signaling and blocks ER stress-induced apoptosis and heart failure. *Nat. Commun.* 8:133. 28743963 10.1038/s41467-017-00171-w PMC5527107

[B50] Zhang D. Wang B. Ma M. Yu K. Zhang Q. Zhang X. (2019). lncRNA HOTAIR protects myocardial infarction rat by sponging miR-519d-3p. *J. Cardiovasc. Transl. Res.* 12 171–183. 10.1007/s12265-018-9839-4 30607799

[B51] Zhang J. C. Xia L. Jiang Y. Wu D. Q. Liu S. C. Zhou X. N. (2019). Effect of lncRNA GAS5 on rats with acute myocardial infarction through regulating miR-21. *Eur. Rev. Med. Pharmacol. Sci.* 23 8573–8579. 31646590 10.26355/eurrev_201910_19173

[B52] Zhang R. Li Y. Liu X. Qin S. Guo B. Chang L. (2020). FOXO3a-mediated long non-coding RNA LINC00261 resists cardiomyocyte hypoxia/reoxygenation injury via targeting miR23b-3p/NRF2 axis. *J. Cell. Mol. Med.* 24 8368–8378. 10.1111/jcmm.15292 32558131 PMC7412708

[B53] Zhang S. Lin X. Li G. Shen X. Niu D. Lu G. (2017). Knockout of Eva1a leads to rapid development of heart failure by impairing autophagy. *Cell Death Dis.* 8:e2586. 10.1038/cddis.2017.17 28151473 PMC5386466

[B54] Zhang Y. Liu T. Meyer C. A. Eeckhoute J. Johnson D. S. Bernstein B. E. (2008). Model-based analysis of ChIP-Seq (MACS). *Genome Biol.* 9:R137. 18798982 10.1186/gb-2008-9-9-r137 PMC2592715

[B55] Zhou L. Zhu Y. Sun D. Zhang Q. (2020). Emerging roles of long non-coding RNAs in the tumor microenvironment. *Int. J. Biol. Sci.* 16 2094–2103. 10.7150/ijbs.44420 32549757 PMC7294937

[B56] Zhu Z. Zhao C. (2019). LncRNA AK139128 promotes cardiomyocyte autophagy and apoptosis in myocardial hypoxia-reoxygenation injury. *Life Sci.* 2:116705. 10.1016/j.lfs.2019.116705 31369757

[B57] Zidan A. Awaisu A. Kheir N. Mahfoud Z. Kaddoura R. AlYafei S. (2016). Impact of a pharmacist-delivered discharge and follow-up intervention for patients with acute coronary syndromes in Qatar: a study protocol for a randomised controlled trial. *BMJ Open* 6:e012141. 10.1136/bmjopen-2016-012141 27864247 PMC5129077

