# Supplementary material for: The Novel Non-coding Transcriptional Regulator Gm18840 Drives Cardiomyocyte Apoptosis in Myocardial Infarction Post Ischemia/Reperfusion
Source: Front Cell Dev Biol. 2021 Jul 12;9:615950. doi: 10.3389/fcell.2021.615950 (PMC8312575; doi:10.3389/fcell.2021.615950)
Supplement: Supplementary file 9 [file Data_Sheet_1.DOCX]

**Supplemental Materials**

**Figure S1**


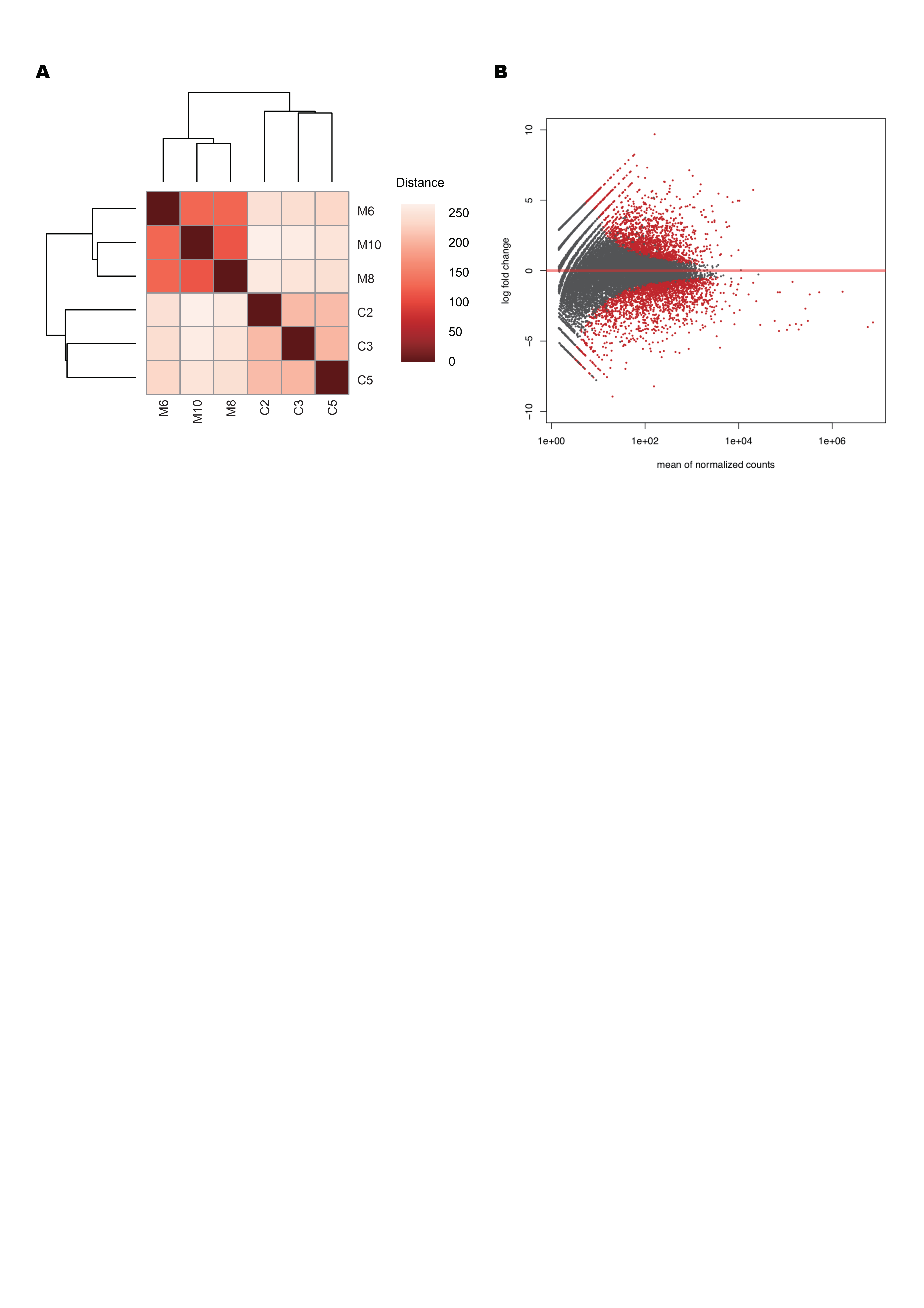


**Figure S1.** The general quality of RNAseq experiments was showed in heatmap (A) and volcano plots (B).

**Figure S2**


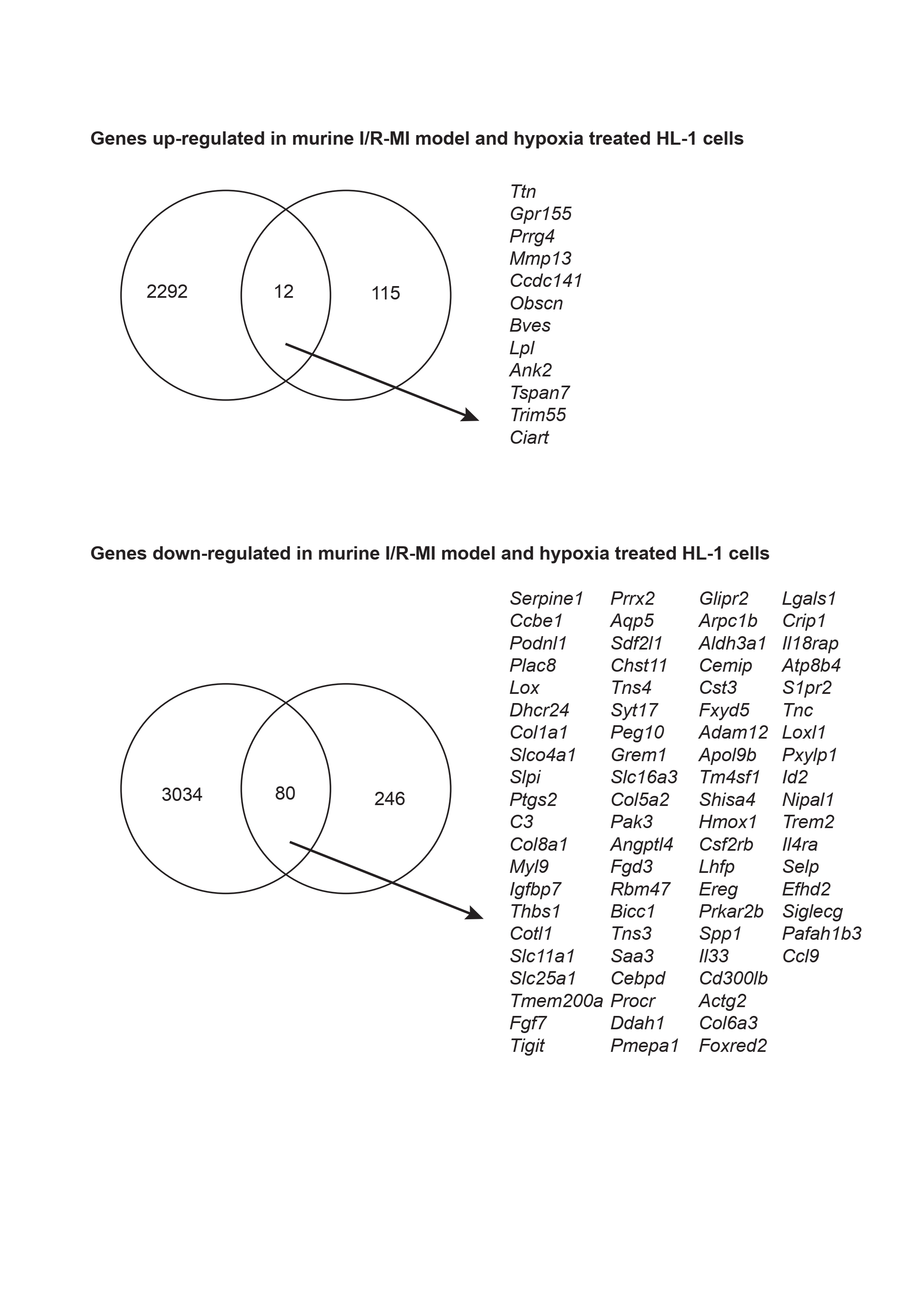


**Figure S2.** Venn diagram showing the common deregulated genes in the different RNA-seq analysis**.**

**Figure S3**


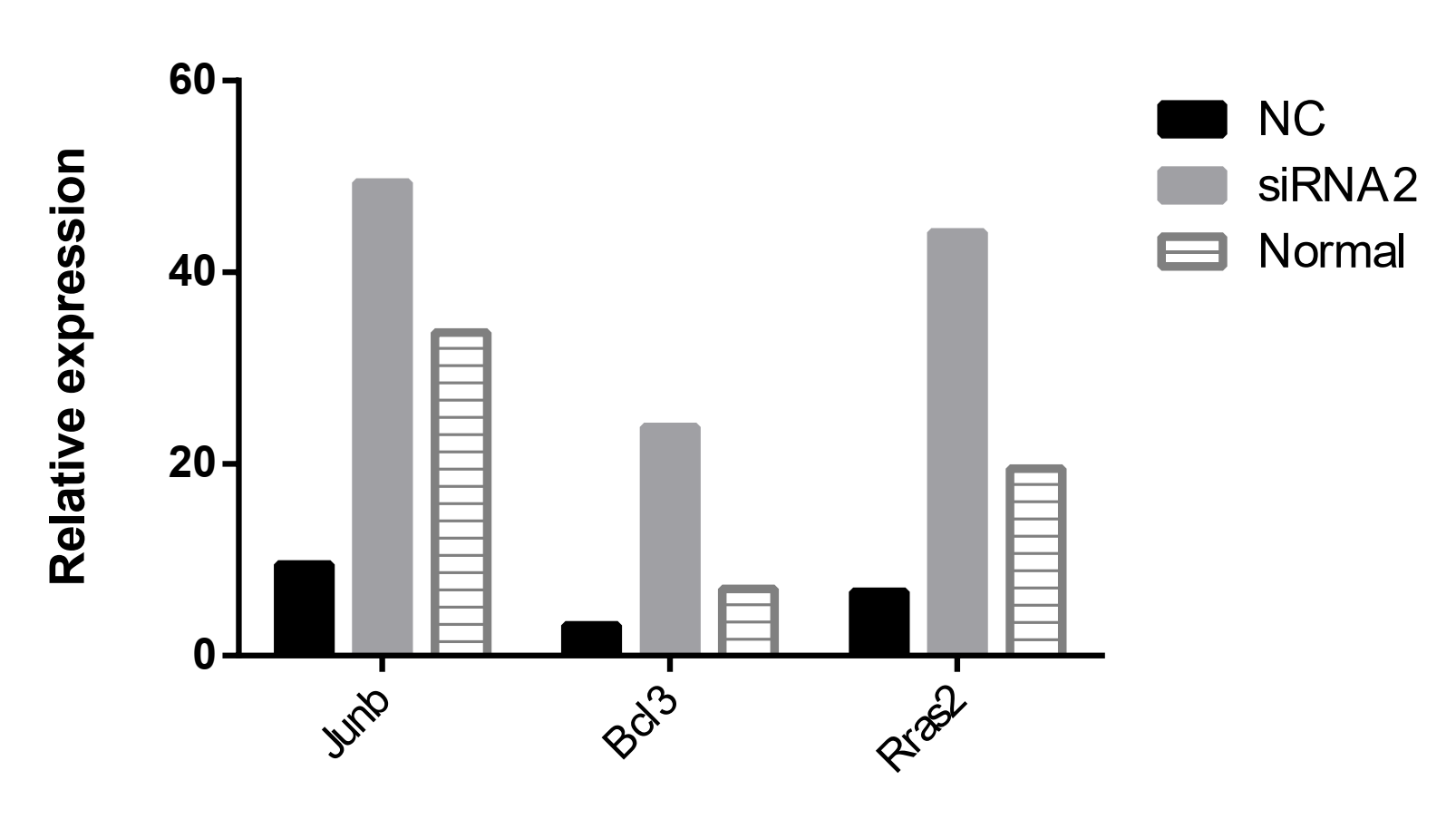


**Figure S3.** The relative expression of Junb, Bcl3 and Rras2 in the condition of hypoxia (NC group), knockdown of Gm18840 under hypoxia (siRNA group), and normoxia (Normal group).
